# Patient Perception of Lower-Limb Straightness at One Week After Unrestricted Kinematically Aligned Total Knee Arthroplasty: Exploring the Concept of “Inherent Straightness”

**DOI:** 10.3390/jpm16020067

**Published:** 2026-01-30

**Authors:** Toshiya Kano, Yoshinori Soda, Kimihiro Inoue, Mitsuhiro Nakamura

**Affiliations:** 1 Department of Orthopedic Surgery, Hiroshima City Hiroshima Citizens Hospital, 7-33 Motomachi, Naka-ku, Hiroshima 730-8518, Japan; kmhr1225@gmail.com; 2 Hiroshima Midorii Joint Replacement Center, Saka Midorii Hospital, 1-28-9 Midorii, Asaminami-ku, Hiroshima 731-0103, Japan; ysoda@ymail.ne.jp (Y.S.); mitsuhiro@m3.dion.ne.jp (M.N.)

**Keywords:** kinematic alignment, total knee arthroplasty, inherent straightness, patient perception, coronal alignment, patient-centered outcomes

## Abstract

**Background/Objectives:** Mechanical neutrality has long been regarded as the principal alignment target in total knee arthroplasty (TKA). However, radiographic neutrality does not necessarily reflect physiological morphology or patient perception. This study aimed to evaluate one-week postoperative patient-perceived lower-limb straightness after unrestricted kinematic alignment (KA) TKA and to examine its relationship with radiographic alignment parameters and functional findings. **Methods:** A total of 175 patients (203 knees) who underwent unrestricted KA-TKA were retrospectively reviewed. Pre- and postoperative radiographs, clinical assessments, and a study-specific, non-validated questionnaire were analyzed one week postoperatively. Patient perception of straightness was assessed using the Straightness Visual Analog Scale (S-VAS) and the Straightness Satisfaction Visual Analog Scale (SS-VAS). Radiographic parameters included the hip–knee–ankle angle (HKAA), the medial proximal tibial angle (MPTA), the mechanical lateral distal femoral angle (mLDFA), the joint line convergence angle (JLCA), and Coronal Plane Alignment of the Knee (CPAK) patterns. Correlative analyses between VAS scores and radiographic and clinical parameters were examined. **Results:** Preoperatively, 85% of knees were perceived as bowed, and all were reported as straight after surgery. Among knees not perceived as bowed preoperatively, 60% were newly perceived as straight postoperatively, while 40% remained perceived as straight. Postoperative satisfaction was high (S-VAS 88.9 ± 11.6; SS-VAS 92.3 ± 12.9). Associations between S-VAS/SS-VAS and HKAA were weak but statistically significant, whereas both showed moderate positive correlations with postoperative knee extension (S-VAS r = 0.54; SS-VAS r = 0.59). **Conclusions:** At one week after surgery, patients tended to perceive lower-limb straightness as being associated with restoration of physiological morphology and improved knee extension rather than with radiographic mechanical neutrality. Patient-perceived straightness reflects an individualized and natural sense of limb alignment (“inherent straightness”) and should be interpreted as an exploratory, patient-centered concept based on an early postoperative test, supporting a personalized framework for alignment evaluation in unrestricted KA-TKA.

## 1. Introduction

Since the late 1970s, mechanical neutrality has been widely regarded as the principal alignment goal in total knee arthroplasty (TKA) for osteoarthritic knees [1,2]. This strategy was proposed to improve implant survival by promoting even load distribution between the medial and lateral compartments. Over time, the neutral mechanical axis has also come to be widely interpreted as representing “normal” or physiologic alignment [3,4,5,6,7,8,9,10]. However, Bellemans et al. [11] demonstrated that a substantial proportion of skeletally mature adults naturally exhibit varus knee morphology, suggesting that ideal alignment may vary among individuals and does not universally fall within the traditional target of 0° ± 3°.

Furthermore, increasing evidence indicates that coronal alignment measured on static radiographs after TKA does not reliably predict dynamic loading during gait [12,13] and shows only weak associations with postoperative functional recovery or long-term implant performance [14,15]. Consequently, attention has shifted toward alternative alignment philosophies. Recent phenotype-based classifications [16,17] have highlighted the wide inter-individual variability in native coronal alignment across populations, supporting the concept that a single radiographic target may be inappropriate for all patients. Howell and colleagues [18,19,20] proposed kinematic alignment (KA), an approach that aims to restore the patient’s native bone morphology and soft-tissue balance, thereby attempting to replicate prearthritic knee conditions. This individualized alignment concept is consistent with the principles of personalized medicine, which emphasize tailoring treatment strategies to each patient’s native anatomy and functional characteristics rather than applying uniform radiographic targets.

To date, no published studies have examined how patients perceive the straightness of their lower limbs after TKA performed using such individualized alignment techniques, and this aspect remains insufficiently understood. In the present study, “patient-perceived straightness” refers to the patient’s subjective visual and proprioceptive impression of lower-limb alignment, rather than an objective radiographic measurement. In the context of KA, which seeks to reproduce each patient’s physiological morphology, it is clinically relevant to clarify whether patients perceive their limbs as bowed or naturally straight after surgery. Understanding this patient-perceived straightness may provide a patient-centered indicator of successful personalized alignment beyond conventional radiographic metrics.

Therefore, the purpose of this study was to evaluate whether patients perceive their lower limbs to be straight following unrestricted KA-TKA and to investigate how this perception relates to patient satisfaction with perceived straightness, coronal alignment parameters, and early functional findings.

## 2. Materials and Methods

### 2.1. Patients

A total of 175 patients (203 knees) who underwent unrestricted kinematically aligned total knee arthroplasty (KA-TKA) at our institution between February 2023 and June 2025 were included. The mean age was 76.7 ± 7.1 years; 36 were male, and 139 were female. All procedures were performed on a single limb per patient; no bilateral cases were included, and therefore all knees were treated as independent observations in the statistical analyses. All patients were of Japanese ethnicity. Patients with a history of osteotomy, fracture, or total hip arthroplasty on the affected side were excluded to avoid extra-articular influences.

### 2.2. Clinical Evaluation

Knee extension and flexion angles were assessed preoperatively and one week postoperatively using a standard goniometer with patients in the supine position. Measurements were obtained passively, with the examiner supporting the limb to ensure neutral rotation. This one-week postoperative assessment represents very early postoperative status and may be influenced by pain, swelling, quadriceps inhibition, and analgesic use, rather than reflecting stable functional recovery. All measurements were performed by a single observer, and no formal intra- or inter-observer reliability testing was conducted.

### 2.3. Radiographic Evaluation

**Figure 1:**
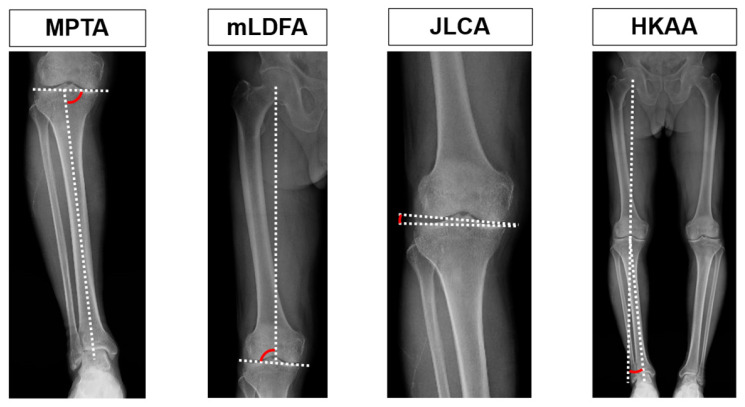
Radiographic definitions of coronal alignment parameters.

Weight-bearing, full-leg standing radiographs were obtained preoperatively and one week postoperatively. Patients stood with their feet 10 cm apart and both patellae facing forward. Radiographic parameters included the medial proximal tibial angle (MPTA), mechanical lateral distal femoral angle (mLDFA), joint line convergence angle (JLCA), and hip–knee–ankle angle (HKAA). Knees were classified according to the Coronal Plane Alignment of the Knee (CPAK) system [17]. Radiographic measurements followed established angle definitions, which are summarized and illustrated in Figure 1 for clarity. All radiographic measurements were performed by a single observer, and measurement reliability was not formally assessed.

Schematic illustration of radiographic measurements used in this study.

The medial proximal tibial angle (MPTA), the mechanical lateral distal femoral angle (mLDFA), the joint line convergence angle (JLCA), and the hip–knee–ankle angle (HKAA) were assessed on weight-bearing radiographs. Dotted lines represent the mechanical axis or joint line, and red arcs indicate the measured angles. For the JLCA, medial opening was defined as positive and lateral opening as negative. For the HKAA, valgus alignment was defined as positive and varus alignment as negative.

### 2.4. Questionnaire Survey

A study-specific questionnaire was administered one week after surgery to evaluate patients’ perceptions of lower-limb straightness. This questionnaire has not undergone formal psychometric validation and should be regarded as an exploratory, study-specific instrument. This point was selected to facilitate comparison between postoperative lower-limb straightness and patients’ recalled preoperative lower-limb shape using standardized full-length standing radiographs. Because the survey relies in part on patients’ recollection of their preoperative limb shape, recall bias cannot be excluded.

The survey consisted of five items:

1. Did you feel that your leg was bowed before the surgery?
2. Has your leg been straightened after the surgery?
3. Are you satisfied with the straightness of your leg?
4. What is your straightness score?
5. What is your score for satisfaction with straightness?

Questions 1–3 were answered with “yes” or “no,” and questions 4–5 were assessed using a visual analog scale (VAS). For the VAS, a score of 0 represented the worst imaginable straightness or satisfaction, and a score of 100 represented the best imaginable straightness or satisfaction.

### 2.5. Surgical Procedures

#### 2.5.1. Distal and Posterior Femoral Resection

The femoral component was aligned using the calipered technique, as previously described [21,22]. After accounting for cartilage wear, distal and posterior resections were performed to match the thickness of the femoral component.

#### 2.5.2. Proximal Tibial Resection

The tibial component was aligned using a combination of the calipered technique [21,22] and the soft-tissue–respecting technique [22,23], both integral to unrestricted KA [24]. With the trial femoral component in place, the knee was extended and the lower leg gently pulled distally. A curved gap gauge was inserted into the tibial osteochondral defect to measure defect thickness. These values were compensated using the calipered technique, and cutting blocks were positioned so that the medial and lateral sides matched the thickness of the tibial component. The posterior slope was aligned to the native medial tibial condyle, and tibial rotation was aligned parallel to the long axis of the lateral tibial condyle. The soft-tissue–respecting technique was then used to confirm parallelism between the tibial resection surface and the distal femoral cut in extension. Minor adjustments were performed when joint-surface deformation or defect evaluation warranted correction. The posterior cruciate ligament was preserved in all patients.

### 2.6. Data Analysis

All values are expressed as means ± standard deviations. Analyses were performed using the R software (version 3.4.1; R Foundation for Statistical Computing, Vienna, Austria). The normality of continuous variables was assessed using the Shapiro–Wilk test. Pre- and postoperative values of range of motion (ROM; extension and flexion), MPTA, mLDFA, JLCA, and HKAA were compared using paired *t*-tests.

Pearson correlation coefficients were calculated to examine associations between straightness VAS (S-VAS) or straightness satisfaction VAS (SS-VAS) and both HKAA and postoperative knee extension. Ninety-five percent confidence intervals (95% CIs) were calculated where appropriate. All correlation analyses were exploratory and unadjusted for potential confounders such as age, sex, or baseline deformity severity. A post hoc power analysis was conducted using G*Power version 3.1 (Heinrich-Heine-University, Düsseldorf, Germany) based on the observed standard deviations. Because this was a post hoc analysis, its interpretive value is limited, and it was not used to support causal inference. Scatter plots displayed regression lines only for correlations of at least moderate strength (r ≥ 0.3) to avoid visual overinterpretation of weak associations.

A two-sided *p*-value < 0.05 was considered statistically significant. No adjustments were made for multiple comparisons because the analyses were exploring in nature.

The datasets analyzed in this study are available from the corresponding author upon reasonable request.

### 2.7. Ethical Considerations

This retrospective observational study was approved by the institutional ethics committee (approval number: 2025-98) and conducted in accordance with the Ethical Guidelines for Medical and Health Research Involving Human Subjects in Japan and the Declaration of Helsinki. All data were obtained from anonymized reviews of routine clinical and radiographic records. Written informed consent was obtained from all patients for the use of their clinical information for research and publication purposes.

Generative artificial intelligence was not used for data generation, data analysis, or interpretation in this study.

## 3. Results

### 3.1. Range of Motion (Short-Term Outcome)

One week after surgery, postoperative knee motion differed significantly from preoperative values. The mean extension angle improved from –16.4° ± 9.8° preoperatively to –5.0° ± 5.7° postoperatively, whereas the mean flexion angle decreased from 119.6° ± 14.8° to 110.2° ± 12.8° (both *p* < 0.01). These findings indicate substantial early correction of the extension deficit, accompanied by a modest reduction in knee flexion. This reduction in knee flexion should be interpreted as an expected finding in the very early postoperative phase, when pain, swelling, and quadriceps inhibition commonly limit active motion, rather than as a true deterioration of functional outcome.

### 3.2. Radiographic Alignment

The mean preoperative and postoperative radiographic parameters are summarized in Table 1. Postoperatively, both MPTA and JLCA significantly increased (both *p* < 0.01), indicating that the joint-line orientation was restored toward a more physiological configuration. The postoperative JLCA was 0° in all cases on standardized standing radiographs. The HKAA also shifted significantly after surgery (*p* < 0.01). In contrast, mLDFA did not differ significantly between time points (*p* = 0.83). Overall, these findings demonstrate that postoperative changes in MPTA and JLCA primarily contributed to the improvement in coronal alignment.

### 3.3. CPAK Classification

**Figure 2:**
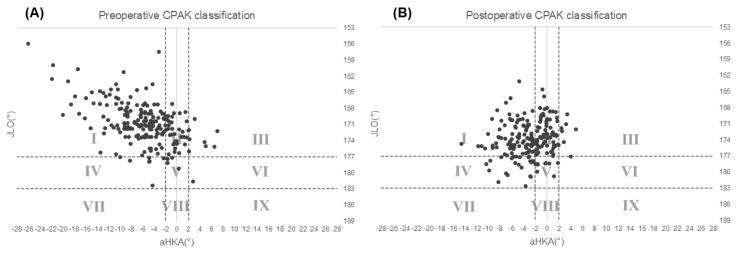
Scatter plots of CPAK classification. (**A**) Preoperative distribution plotted by arithmetic hip–knee–ankle angle (aHKA, *x*-axis) and joint line obliquity (JLO, *y*-axis). (**B**) Postoperative distribution. Dotted lines indicate the Coronal Plane Alignment of the Knee (CPAK) classification thresholds of aHKA = –2° to 2° and JLO = 177° to 183°.

The distribution of CPAK types before and after surgery is shown in Table 2 and Figure 2. Preoperatively, most knees were classified as Type I (150 knees, 74%), followed by Type II (35 knees, 17%). Postoperatively, the proportion of Type I knees decreased to 44% (90 knees), whereas the proportion of Type II knees increased to 36% (72 knees), and Type IV increased to 12% (24 knees). Overall, the percentage of neutral alignment types (II, V, and VIII) increased from 19% preoperatively to 40% postoperatively, indicating a redistribution of CPAK phenotypes after KA-TKA rather than an intentional shift toward radiographic neutrality.

### 3.4. Questionnaire Survey

**Figure 3:**
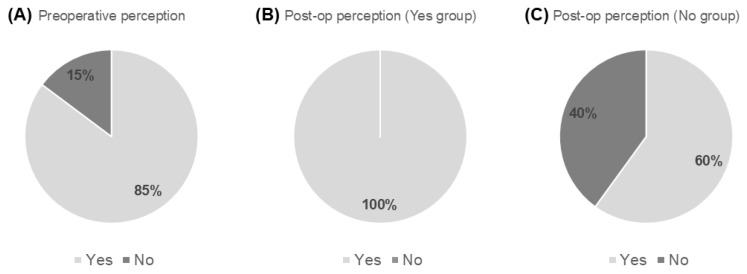
Patient-reported perception of lower-limb alignment. (**A**) Response to the question: “Did you feel that your leg was bowed before the surgery?” (*n* = 203). (**B**) Response to the question: “Has your leg been straightened after the surgery?” among patients who preoperatively perceived their legs as bowed (*n* = 173). (**C**) Response to the question: “Has your leg been straightened after the surgery?” among patients who preoperatively did not perceive their legs as bowed (*n* = 30).

Patient responses are summarized in Figure 3. Preoperatively, 173 of 203 knees (85%) were perceived as bowed. All of these knees were reported as straight after surgery. Among knees not perceived as bowed preoperatively, 60% were newly perceived as straight postoperatively, while 40% continued to perceive their limbs as straight. All patients expressed satisfaction with their postoperative limb straightness.

### 3.5. VAS Scores

The mean postoperative S-VAS was 88.9 ± 11.6 (95% CI: 87.3–90.5), and the SS-VAS was 92.3 ± 12.9 (95% CI: 90.5–94.1). Both distributions skewed toward the upper end of the scale, indicating that patients generally perceived their limbs as nearly straight and were highly satisfied. The post hoc power analysis showed that, with 203 knees and a standard deviation of 11.6, the study had 80% power to detect a 2.3-point difference in the VAS.

### 3.6. Correlations

**Figure 4:**
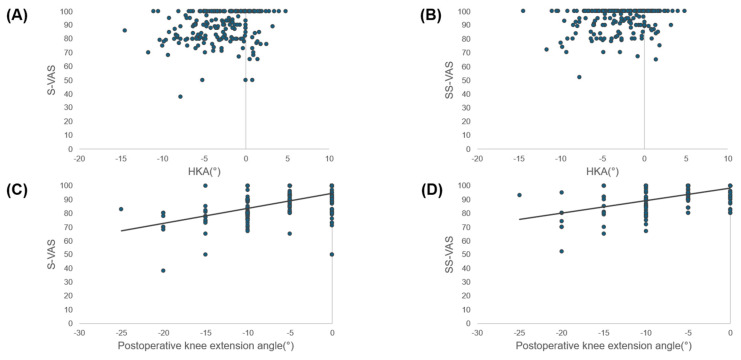
Relationships between patient-reported straightness scores and radiographic/clinical parameters. Scatter plots showing the relationships between straightness visual analog scale (S-VAS) or satisfaction with straightness VAS (SS-VAS) and radiographic/clinical parameters. Regression lines are shown only for correlations with r ≥ 0.3. (**A**) S-VAS versus hip–knee–ankle angle (HKA): weak correlation (r = 0.186, 95% CI 0.049–0.315, *p* = 0.008). (**B**) S-VAS versus postoperative knee extension angle: moderate correlation (r = 0.535, 95% CI 0.429–0.626, *p* < 0.001). (**C**) SS-VAS versus HKA: weak correlation (r = 0.164, 95% CI 0.027–0.295, *p* = 0.019). (**D**) SS-VAS versus postoperative knee extension angle: moderate correlation (r = 0.586, 95% CI 0.487–0.669, *p* < 0.001).

Correlation analyses revealed statistically significant but weak associations between S-VAS and postoperative HKAA (r = 0.186, 95% CI 0.049–0.315, *p* = 0.008) and between SS-VAS and HKAA (r = 0.164, 95% CI 0.027–0.295, *p* = 0.019). However, the magnitude of these correlations was small and below the predefined threshold for moderate association (r ≥ 0.3), and therefore they were considered of limited clinical relevance. In contrast, both indices demonstrated significant positive correlations with postoperative knee extension (S-VAS: r = 0.54, 95% CI 0.43–0.63, *p* < 0.001; SS-VAS: r = 0.59, 95% CI 0.49–0.67, *p* < 0.001) (Figure 4). These findings indicate that patient-perceived straightness and satisfaction were more closely related to functional knee extension than to radiographic alignment.

## 4. Discussion

The most important finding of this study was that patients were satisfied with the perceived straightness of their legs after KA-TKA. This finding highlights a patient-specific and individualized outcome that cannot be fully captured by conventional radiographic alignment targets alone. Many patients in this cohort exhibited femoral cartilage defects with preserved mLDFA, whereas tibial bone defects were common. Consequently, correction of the MPTA led to improvements in overall limb alignment. When these bony and cartilaginous defects were restored, the collapsed articular surface on the osteoarthritic side opened, and the JLCA approached 0°. Because KA-TKA aims to reproduce the prearthritic joint state rather than implanting components into a deformed joint, postoperative changes in alignment and corresponding shifts in CPAK classification should be interpreted as the correction of bone defects rather than unintended deviations in alignment. Patients perceived these alignment changes as their legs becoming straight and expressed high satisfaction with perceived straightness in the coronal plane. In addition, sensory perception of straightness was influenced by knee extension; when residual extension deficits persisted, satisfaction tended to decrease. This distinction between radiographic correction and perceptual straightness provides a useful context for reconsidering the traditional emphasis on mechanical neutrality.

Traditionally, achieving mechanical neutrality (HKAA = 0°) has been regarded as the standard alignment target in TKA. However, the present findings suggest that this radiographic benchmark does not necessarily correspond to patients’ subjective perception of straightness, indicating a potential discrepancy between objective alignment indices and patient-reported experience. Recent concepts in KA [25] and personalized TKA [26] have further emphasized that restoration of function and patient satisfaction do not require mechanical neutrality and should instead be guided by each patient’s native anatomy and kinematic characteristics. Moreover, the assumption that a mechanically neutral axis is universally optimal may overlook inherent morphological diversity across populations, which reflects long-term evolutionary and developmental influences [27,28,29]. From the perspective of personalized medicine, these findings suggest that a single alignment target may be inappropriate for all patients.

Previous epidemiological studies [30,31] in Asian populations have demonstrated a high prevalence of varus alignment, with reports ranging from one-third to nearly two-thirds of arthritic knees. Comparative analyses further reported that Japanese individuals show greater varus tendencies than Caucasian individuals do [32]. Other investigations [33,34] have confirmed that varus alignment is particularly common among Japanese patients with osteoarthritis, and that progressive varus deformity occurs with advancing age, especially in women [35].

A systematic review of the CPAK classification [36] revealed significant differences in arthritic knees between racial groups but not in healthy knees. KA-TKA, which aims to restore the prearthritic morphology, is therefore expected to modify CPAK categories and generate favorable postoperative limb morphology even in Asian populations. In the present cohort, the proportion of CPAK type I knees was particularly high, representing a distinctive distribution in the global context. Despite these baseline characteristics, patients reported excellent postoperative satisfaction with perceived straightness, supporting the ability of KA-TKA to restore physiological morphology in a manner consistent with patient perception.

Changes in CPAK phenotype following TKA have been widely documented. Studies of mechanical alignment TKA [37] reported that alterations in CPAK category predicted inferior outcomes in KOOS-12 and FJS-12 scores. Simulation analyses [38] further suggested that, in certain phenotypes (e.g., CPAK type I), KA may achieve more favorable soft-tissue balance than MA. Collectively, these findings highlight the importance of respecting an individual’s baseline bone morphology and ligament balance, factors that likely contributed to the natural sense of straightness reported by patients in the present study.

From a broader perspective, these findings have notable clinical implications. For decades, surgeons have equated HKAA = 0° with a “straight leg,” shaping both surgical philosophy and patient expectations. This concept has been reinforced by the notion of “constitutional varus,” derived from radiographic surveys reporting an average HKAA of approximately 3° varus in healthy adults. The present results suggest that, at least in the very early postoperative phase, patients’ perception of straightness is influenced primarily by the restoration of native morphology and adequate knee extension rather than by achieving radiographic neutrality. This patient-centered alignment can be described as an “inherent straightness”—an alignment that feels physiologically natural to the individual. At present, inherent straightness should be regarded as an exploratory, hypothesis-generating concept rather than a validated alternative to radiographic alignment targets. Distinguishing inherent straightness from radiographic neutrality may help refine current concepts of alignment and underscores the value of individualized anatomical reconstruction in KA-TKA.

## 5. Limitations

This study has several limitations that should be acknowledged. First, it was retrospective and conducted at a single center, which may restrict the generalizability of the findings. Second, patient perception of straightness was assessed using a study-specific VAS instrument. Because this instrument was developed for the purpose of this study and has not undergone formal psychometric validation, the findings related to patient perception should be interpreted within this methodological limitation. Accordingly, the S-VAS and SS-VAS should be regarded as exploratory tools, and their results should not be interpreted as validated clinical outcome measures. In addition, the uniformly high satisfaction and the skewed distributions toward the upper end suggest a possible ceiling effect that may limit the discriminative capacity of the VAS at this early point. Third, all evaluations were performed one week postoperatively. This time point was intentionally selected because patients were considered more likely to recall their preoperative limb shape and to compare it with their postoperative lower-limb straightness, and because standardized full-length standing radiographs could be reliably obtained at this stage. However, this very early postoperative phase is strongly influenced by swelling, pain, analgesic use, and reduced mobility, all of which may interfere with accurate limb awareness and sensory perception. In addition, the uniformly high satisfaction observed at this early time point may reflect a ceiling effect, as well as possible gratitude bias and an analgesia-driven early postoperative “relief” effect, which may transiently inflate patient-reported satisfaction. Patient recall of preoperative limb morphology is likely to diminish over time, and attention to limb shape tends to decrease as functional recovery progresses. Therefore, the findings of this study reflect very early postoperative perception and cannot be directly extrapolated to longer-term perceptual or functional outcomes. Fourth, radiographic measurements and ROM assessments were performed by a single observer, and intra- or inter-observer reliability was not assessed. Fifth, correlation analyses were exploratory and based on linear assumptions; therefore, weak correlations should be interpreted with caution. In particular, the observed associations between knee extension and perception scores should be regarded as correlations only, and no causal relationship can be inferred from the present data. Finally, although KA-TKA aims to restore each patient’s native morphology, it remains uncertain to what extent the reconstructed joint surface replicates the true prearthritic anatomy. Ongoing morphological investigations may help clarify this issue. Nevertheless, the central finding of this study remains consistent: patients reported a high degree of satisfaction with perceived straightness of the lower limb after unrestricted KA-TKA, regardless of their postoperative radiographic alignment.

## 6. Conclusions

Patients who underwent unrestricted KA-TKA were highly satisfied with the perceived straightness of their legs, even when postoperative alignment did not reach mechanical neutrality (HKAA = 0°). Satisfaction was more closely associated with the restoration of knee extension and physiological morphology than with radiographic coronal alignment. These findings indicate that the alignment patients perceive as “straight” reflects an individualized and natural sense of limb alignment—an “inherent straightness.” In the context of this early postoperative, single-center, exploratory study using a non-validated instrument, inherent straightness should be regarded as a hypothesis-generating, patient-centered concept rather than a validated alternative to radiographic alignment targets. Recognizing inherent straightness as a meaningful patient-centered outcome may help refine current concepts of alignment in TKA and complement traditional radiographic metrics.

## Figures and Tables

**Table 1:** Changes in radiographic alignment parameters before and after surgery.

Parameter	Preoperative (Mean ± SD)	Postoperative (Mean ± SD)	*p*-Value
MPTA (°)	82.4 ± 4.1	85.4 ± 2.2	<0.001
mLDFA (°)	88.4 ± 2.3	88.4 ± 2.6	0.83
JLCA (°)	−4.9 ± 4.2	0 ± 0	<0.001
HKAA (°)	−10.8 ± 7.9	−3.0 ± 3.4	<0.001

Abbreviations: MPTA = medial proximal tibial angle; mLDFA = mechanical lateral distal femoral angle; JLCA = joint line convergence angle; HKAA = hip–knee–ankle angle.

**Table 2:** Distribution of CPAK classification before and after surgery.

CPAK Type	Preoperative, *n* (%)	Postoperative, *n* (%)
Type I	150 (74)	90 (44)
Type II	35 (17)	72 (36)
Type III	6 (3)	6 (3)
Type IV	7 (3)	24 (12)
Type V	4 (2)	10 (5)
Type VI	1 (<1)	1 (<1)

Abbreviation: CPAK = Coronal Plane Alignment of the Knee.

## Data Availability

The datasets analyzed during the current study are available from the corresponding author upon reasonable request.

## Abbreviations

The following abbreviations are used in this manuscript:

CPAK	Coronal Plane Alignment of the Knee
HKAA	Hip–Knee–Ankle Angle
JLCA	Joint Line Convergence Angle
KA	Kinematic Alignment
KA-TKA	Kinematically Aligned Total Knee Arthroplasty
MPTA	Medial Proximal Tibial Angle
mLDFA	Mechanical Lateral Distal Femoral Angle
ROM	Range of Motion
S-VAS	Straightness Visual Analog Scale
SS-VAS	Straightness Satisfaction Visual Analog Scale
TKA	Total Knee Arthroplasty
VAS	Visual Analog Scale
